# 1′,1′′-Dimethyl-4′-(4-methyl­phen­yl)di­spiro­[11*H*-indeno­[1,2-b]quinoxaline-11,2′-pyrrolidine-3′,3′′-piperidin]-4′′-one

**DOI:** 10.1107/S1600536813022964

**Published:** 2013-08-23

**Authors:** R. A. Nagalakshmi, J. Suresh, K. Malathi, R. Ranjith Kumar, P. L. N. Lakshman

**Affiliations:** a Department of Physics, The Madura College, Madurai 625 011, India; b Department of Organic Chemistry, School of Chemistry, Madurai Kamaraj University, Madurai 625 021, India; c Department of Food Science and Technology, University of Ruhuna, Mapalana, Kamburupitiya 81100, Sri Lanka

## Abstract

In the title compound, C_31_H_30_N_4_O, the central pyrrolidine ring adopts an envelope conformation with the methyl­ene C atom being the flap. The quinoxaline and indane rings are each planar, having r.m.s. deviations of 0.030 and 0.050 Å, respectively. The pyrrolidine ring mean plane forms dihedral angles of 88.25 (1) and 83.76 (1)° with the quinoxaline and indane rings, respectively. Intra­molecular C—H⋯O and C—H⋯N inter­actions are observed. In the crystal, C—H⋯π inter­actions lead to helical supra­molecular chains along the *b-*axis direction.

## Related literature

For the importance of pyrrolidine compounds, see: Witherup *et al.* (1995 ▶). For the importance of heterocycles with piperidine sub-structures, see: El-Subbagh *et al.* (2000 ▶); Dimmock *et al.* (2001 ▶); Lee *et al.* (2001 ▶). For additional conformation analysis, see: Cremer & Pople (1975 ▶).
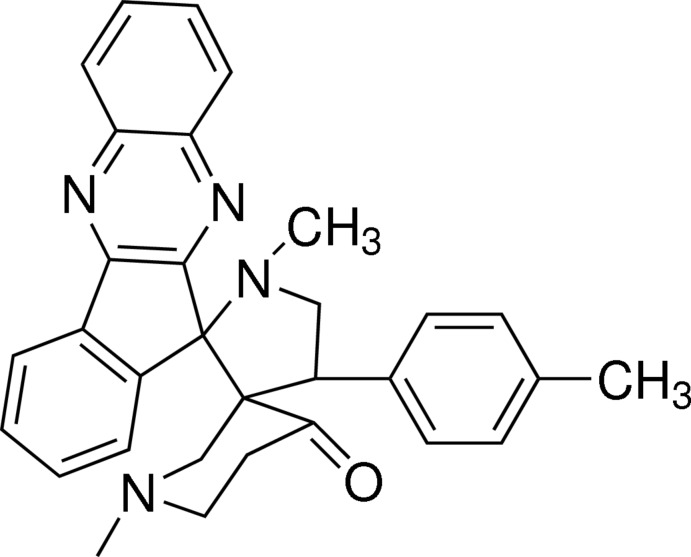


## Experimental

### 

#### Crystal data

C_31_H_30_N_4_O
*M* *_r_* = 474.59
Monoclinic, 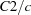

*a* = 22.3183 (7) Å
*b* = 14.4411 (5) Å
*c* = 17.2474 (6) Å
β = 116.547 (2)°
*V* = 4972.8 (3) Å^3^
*Z* = 8
Mo *K*α radiation
μ = 0.08 mm^−1^
*T* = 293 K
0.21 × 0.19 × 0.18 mm

#### Data collection

Bruker Kappa APEXII diffractometer
Absorption correction: multi-scan (*SADABS*; Sheldrick, 1996 ▶) *T*_min_ = 0.967, *T*_max_ = 0.974
22517 measured reflections
4550 independent reflections
3034 reflections with *I* > 2σ(*I*)
*R*_int_ = 0.031

#### Refinement

*R*[*F* ^2^ > 2σ(*F* ^2^)] = 0.042
*wR*(*F* ^2^) = 0.124
*S* = 1.04
4550 reflections
327 parameters
H-atom parameters constrained
Δρ_max_ = 0.18 e Å^−3^
Δρ_min_ = −0.12 e Å^−3^

### 

Data collection: *APEX2* (Bruker, 2004 ▶); cell refinement: *SAINT* (Bruker, 2004 ▶); data reduction: *SAINT*; program(s) used to solve structure: *SHELXS97* (Sheldrick, 2008 ▶); program(s) used to refine structure: *SHELXL97* (Sheldrick, 2008 ▶); molecular graphics: *PLATON* (Spek, 2009 ▶); software used to prepare material for publication: *SHELXL97*.

## Supplementary Material

Crystal structure: contains datablock(s) global, I. DOI: 10.1107/S1600536813022964/tk5247sup1.cif

Structure factors: contains datablock(s) I. DOI: 10.1107/S1600536813022964/tk5247Isup2.hkl

Additional supplementary materials:  crystallographic information; 3D view; checkCIF report

## Figures and Tables

**Table 1:** Hydrogen-bond geometry (Å, °)

*D*—H⋯*A*	*D*—H	H⋯*A*	*D*⋯*A*	*D*—H⋯*A*
C3—H3⋯O1	0.98	2.36	2.804 (2)	107
C41—H41*B*⋯N3	0.97	2.39	2.980 (2)	119
C11—H11⋯*Cg*1^i^	0.93	2.94	3.692 (2)	139

Symmetry code: (i) 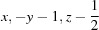.

**Fig. 1.**
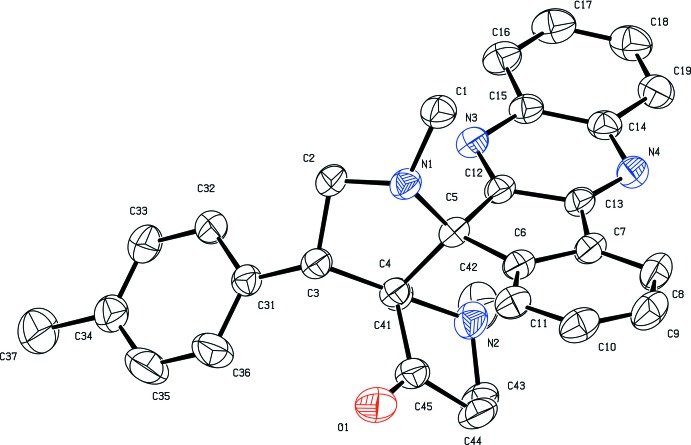
The molecular structure of (I), showing 30% probability displacement ellipsoids and the atom-numbering scheme. H-atoms are omitted for clarity.

**Fig. 2.**
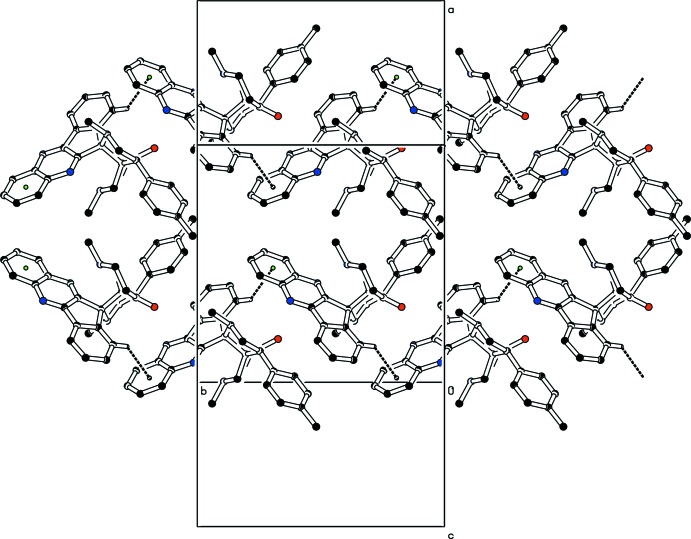
The partial packing diagram showing C—H···π interactions as dashed lines. All H-atoms are omitted for clarity except for the H atom involved in the intermolecular interaction.

## supplementary crystallographic information

### 1. Comment

Pyrrolidine-containing compounds are of significant importance because of their biological activities and widespread employment in catalysis (Witherup *et al.*, 1995). Heterocycles with piperidine sub-structures display important biological activities, such as cytotoxic (El-Subbagh *et al.*, 2000) and anti-cancer (Dimmock *et al.*, 2001) besides being useful as synthons in the construction of alkaloid natural products (Lee *et al.*, 2001). The high medicinal value of these compounds, in conjunction with our research interests, prompted us to synthesize and report the X-ray studies of the title compound.

In the title compound (Fig. 1), the central pyrrolidine ring is an envelope on C2 with asymmetry parameters ΔC_s_(C2) = 7.1 (2)° and puckering parameters q_2_ = 0.4303 (18) Å and φ_2_ = 211.5 (2)° (Cremer & Pople, 1975). The quinoxaline and the indane group forms dihedral angles of 88.25 (1) and 83.76 (1)°, respectively, with the central pyrrolidine ring. The quinoxaline ring system (C12—C17/N3,N4) is planar, with r.m.s. deviation = 0.030 Å. The indane group is also planar with r.m.s deviation = 0.050 Å. The dihedral angle between the mean planes of the fused quinoxaline and the indane groups is 8.43 (1)°, indicate that the fused rings is slightly folded about the C12—C13 bond. The six-membered ring, N2/C41—C45, exhibits a twisted chair conformation, as indicated by the asymmetry parameters ΔC_s_(N2) = 7.58 (16)°, ΔC_s_(C45) = 7.58 (16)° and with the puckering parameters Q = 0.558 (2) Å, Θ = 164.1 (2)° and Φ = 207.1 (8)°. The torsion angle C4—C42—N2—C42 is -167.62 (16)° and corresponds to an antiperiplanar conformation. The sum of bond angles around N1(339.9°) and N2 (331.4°) indicate the atoms N1 and N2 are each in a pyramidal geometry. Weak intramolecular C—H···O, N interactions are observed (Table 1). In the crystal structure, C—H···π interactions, involving the benzene ring C14···C19, lead to the helical supramolecular chains along the *b* axis, as shown in Fig. 2.

### 2. Experimental

A mixture of 1-methyl-3-[*E*-(4-methylphenyl)methylidene]tetrahydro-2(1*H*)- pyridinone (1 mmol), ninhydrin (1 mmol), *o*-phenylenediamine (1 mmol) and sarcosine (1 mmol) in methanol was refluxed for 3–4 h. After completion of the reaction as indicated by TLC the reaction mixture was poured into cold water. The solid precipitate obtained was filtered and dried. The product was purified by column chromatography using petroleum ether:ethylacetate mixture (90:10 *v*/*v*). Suitable crystals were obtained by recrystallizing the product from methanol. Yield: 37%, M. pt: 498–500 K.

### 3. Refinement

H atoms were placed at calculated positions and allowed to ride on their carrier atoms with C—H = 0.93–0.98 Å, and with *U*_iso_ = 1.2*U*_eq_(C) for CH_2_ and CH groups, and *U*_iso_ = 1.5*U*_eq_(C) for CH_3_ groups. The (-1 1 1) reflection was affected by the beam-stop and was removed from the final refinement.

### Crystal data

**Table:**

C_31_H_30_N_4_O	*F*(000) = 2016
*M**_r_* = 474.59	*D*_x_ = 1.268 Mg m^−^^3^
Monoclinic, *C*2/*c*	Mo *K*α radiation, λ = 0.71073 Å
Hall symbol: -C 2yc	Cell parameters from 2000 reflections
*a* = 22.3183 (7) Å	θ = 2–31°
*b* = 14.4411 (5) Å	µ = 0.08 mm^−^^1^
*c* = 17.2474 (6) Å	*T* = 293 K
β = 116.547 (2)°	Block, green
*V* = 4972.8 (3) Å^3^	0.21 × 0.19 × 0.18 mm
*Z* = 8	

### Data collection

**Table:**

Bruker Kappa APEXII diffractometer	4550 independent reflections
Radiation source: fine-focus sealed tube	3034 reflections with *I* > 2σ(*I*)
Graphite monochromator	*R*_int_ = 0.031
Detector resolution: 0 pixels mm^-1^	θ_max_ = 25.4°, θ_min_ = 2.0°
ω & \j scans	*h* = −26→25
Absorption correction: multi-scan (*SADABS*; Sheldrick, 1996)	*k* = −17→17
*T*_min_ = 0.967, *T*_max_ = 0.974	*l* = −20→20
22517 measured reflections	

### Refinement

**Table:**

Refinement on *F*^2^	Secondary atom site location: difference Fourier map
Least-squares matrix: full	Hydrogen site location: inferred from neighbouring sites
*R*[*F*^2^ > 2σ(*F*^2^)] = 0.042	H-atom parameters constrained
*wR*(*F*^2^) = 0.124	*w* = 1/[σ^2^(*F*_o_^2^) + (0.0634*P*)^2^ + 0.8964*P*] where *P* = (*F*_o_^2^ + 2*F*_c_^2^)/3
*S* = 1.04	(Δ/σ)_max_ < 0.001
4550 reflections	Δρ_max_ = 0.18 e Å^−^^3^
327 parameters	Δρ_min_ = −0.12 e Å^−^^3^
0 restraints	Extinction correction: *SHELXL97* (Sheldrick, 2008), Fc^*^=kFc[1+0.001xFc^2^λ^3^/sin(2θ)]^-1/4^
Primary atom site location: structure-invariant direct methods	Extinction coefficient: 0.0021 (3)

### Special details

**Table:**

Geometry. All e.s.d.'s (except the e.s.d. in the dihedral angle between two l.s. planes) are estimated using the full covariance matrix. The cell e.s.d.'s are taken into account individually in the estimation of e.s.d.'s in distances, angles and torsion angles; correlations between e.s.d.'s in cell parameters are only used when they are defined by crystal symmetry. An approximate (isotropic) treatment of cell e.s.d.'s is used for estimating e.s.d.'s involving l.s. planes.
Refinement. Refinement of *F*^2^ against ALL reflections. The weighted *R*-factor *wR* and goodness of fit *S* are based on *F*^2^, conventional *R*-factors *R* are based on *F*, with *F* set to zero for negative *F*^2^. The threshold expression of *F*^2^ > σ (*F*^2^) is used only for calculating *R*-factors(gt) *etc*. and is not relevant to the choice of reflections for refinement. *R*-factors based on *F*.^2^ are statistically about twice as large as those based on *F*, and *R*-factors based on ALL data will be even larger.

### Fractional atomic coordinates and isotropic or equivalent isotropic displacement parameters (Å^2^)

**Table:**

	*x*	*y*	*z*	*U*_iso_*/*U*_eq_	
C1	0.19283 (9)	0.44011 (12)	0.17083 (12)	0.0662 (5)	
H1A	0.1556	0.4180	0.1193	0.099*	
H1B	0.2142	0.4902	0.1562	0.099*	
H1C	0.1772	0.4614	0.2113	0.099*	
C2	0.26183 (9)	0.31684 (11)	0.15307 (11)	0.0566 (4)	
H2A	0.2837	0.3582	0.1293	0.068*	
H2B	0.2245	0.2865	0.1060	0.068*	
C3	0.31046 (8)	0.24719 (10)	0.21449 (10)	0.0515 (4)	
H3	0.2835	0.2026	0.2282	0.062*	
C4	0.35031 (8)	0.30452 (10)	0.29852 (10)	0.0497 (4)	
C5	0.30037 (8)	0.38860 (10)	0.28912 (10)	0.0494 (4)	
C6	0.28097 (8)	0.40162 (11)	0.36293 (11)	0.0522 (4)	
C7	0.30097 (8)	0.48735 (12)	0.40302 (10)	0.0548 (4)	
C8	0.28659 (10)	0.51283 (14)	0.47011 (11)	0.0694 (5)	
H8	0.3003	0.5699	0.4972	0.083*	
C9	0.25172 (11)	0.45217 (16)	0.49585 (13)	0.0780 (6)	
H9	0.2422	0.4680	0.5414	0.094*	
C10	0.23070 (10)	0.36849 (15)	0.45534 (12)	0.0714 (5)	
H10	0.2065	0.3288	0.4733	0.086*	
C11	0.24484 (9)	0.34188 (13)	0.38817 (11)	0.0615 (5)	
H11	0.2303	0.2851	0.3607	0.074*	
C12	0.32976 (8)	0.48350 (10)	0.28851 (10)	0.0489 (4)	
C13	0.33176 (8)	0.53940 (11)	0.35792 (10)	0.0510 (4)	
C14	0.37575 (8)	0.65677 (11)	0.31451 (11)	0.0554 (4)	
C15	0.37010 (8)	0.60413 (11)	0.24282 (11)	0.0540 (4)	
C16	0.39018 (9)	0.64307 (13)	0.18372 (12)	0.0653 (5)	
H16	0.3863	0.6091	0.1359	0.078*	
C17	0.41534 (10)	0.73044 (14)	0.19630 (15)	0.0768 (6)	
H17	0.4282	0.7561	0.1566	0.092*	
C18	0.42209 (10)	0.78177 (14)	0.26775 (15)	0.0787 (6)	
H18	0.4400	0.8412	0.2759	0.094*	
C19	0.40282 (10)	0.74624 (12)	0.32589 (13)	0.0688 (5)	
H19	0.4076	0.7814	0.3735	0.083*	
C31	0.35014 (8)	0.19158 (10)	0.17934 (11)	0.0518 (4)	
C32	0.35959 (11)	0.21834 (12)	0.10931 (12)	0.0686 (5)	
H32	0.3428	0.2751	0.0832	0.082*	
C33	0.39341 (11)	0.16312 (14)	0.07647 (13)	0.0769 (6)	
H33	0.3988	0.1840	0.0290	0.092*	
C34	0.41901 (11)	0.07944 (15)	0.11128 (14)	0.0785 (6)	
C35	0.40965 (15)	0.05333 (16)	0.18085 (18)	0.1093 (9)	
H35	0.4264	−0.0036	0.2066	0.131*	
C36	0.37650 (13)	0.10758 (14)	0.21450 (16)	0.0908 (7)	
H36	0.3718	0.0866	0.2625	0.109*	
C37	0.45532 (15)	0.0190 (2)	0.07414 (18)	0.1260 (10)	
H37A	0.4938	−0.0084	0.1206	0.189*	
H37B	0.4694	0.0560	0.0390	0.189*	
H37C	0.4258	−0.0290	0.0393	0.189*	
C41	0.41760 (8)	0.33778 (12)	0.30566 (11)	0.0563 (4)	
H41A	0.4444	0.2846	0.3062	0.068*	
H41B	0.4100	0.3751	0.2554	0.068*	
C42	0.51060 (10)	0.44002 (16)	0.38092 (16)	0.0959 (7)	
H42A	0.4946	0.4798	0.3311	0.144*	
H42B	0.5410	0.3953	0.3771	0.144*	
H42C	0.5334	0.4763	0.4325	0.144*	
C43	0.47597 (11)	0.33179 (16)	0.45900 (13)	0.0862 (7)	
H43A	0.5019	0.3672	0.5112	0.103*	
H43B	0.5045	0.2835	0.4547	0.103*	
C44	0.41635 (11)	0.28845 (15)	0.46469 (12)	0.0812 (6)	
H44A	0.4321	0.2401	0.5083	0.097*	
H44B	0.3944	0.3352	0.4834	0.097*	
C45	0.36603 (10)	0.24775 (13)	0.38038 (12)	0.0633 (5)	
N1	0.24057 (7)	0.36540 (9)	0.20961 (9)	0.0539 (4)	
N2	0.45411 (7)	0.39221 (11)	0.38393 (10)	0.0678 (4)	
N3	0.34651 (7)	0.51439 (9)	0.22985 (9)	0.0539 (4)	
N4	0.35471 (7)	0.62400 (9)	0.37323 (9)	0.0581 (4)	
O1	0.34059 (9)	0.17381 (10)	0.37835 (10)	0.0914 (5)	

### Atomic displacement parameters (Å^2^)

**Table:**

	*U*^11^	*U*^22^	*U*^33^	*U*^12^	*U*^13^	*U*^23^
C1	0.0577 (11)	0.0755 (12)	0.0587 (12)	0.0110 (9)	0.0200 (10)	0.0029 (9)
C2	0.0560 (10)	0.0630 (10)	0.0429 (10)	−0.0017 (8)	0.0151 (9)	−0.0044 (8)
C3	0.0545 (10)	0.0525 (9)	0.0458 (10)	−0.0054 (8)	0.0207 (8)	0.0003 (7)
C4	0.0529 (10)	0.0545 (9)	0.0402 (9)	0.0012 (8)	0.0194 (8)	0.0015 (7)
C5	0.0488 (9)	0.0563 (9)	0.0410 (9)	−0.0019 (7)	0.0183 (8)	−0.0003 (7)
C6	0.0484 (10)	0.0637 (10)	0.0434 (10)	0.0044 (8)	0.0194 (8)	0.0042 (8)
C7	0.0516 (10)	0.0692 (11)	0.0409 (10)	0.0051 (8)	0.0182 (8)	0.0006 (8)
C8	0.0735 (13)	0.0864 (13)	0.0489 (11)	0.0002 (11)	0.0278 (11)	−0.0106 (9)
C9	0.0778 (14)	0.1113 (16)	0.0546 (12)	0.0052 (13)	0.0382 (12)	−0.0022 (11)
C10	0.0655 (13)	0.0987 (15)	0.0574 (12)	0.0015 (11)	0.0340 (11)	0.0109 (11)
C11	0.0584 (11)	0.0710 (11)	0.0551 (11)	−0.0014 (9)	0.0253 (10)	0.0046 (9)
C12	0.0459 (9)	0.0567 (9)	0.0388 (9)	0.0025 (7)	0.0142 (8)	0.0017 (7)
C13	0.0478 (9)	0.0570 (9)	0.0401 (9)	0.0044 (8)	0.0125 (8)	−0.0005 (7)
C14	0.0460 (10)	0.0554 (10)	0.0531 (11)	0.0040 (8)	0.0116 (9)	0.0055 (8)
C15	0.0452 (9)	0.0595 (10)	0.0488 (10)	0.0002 (8)	0.0135 (8)	0.0054 (8)
C16	0.0568 (11)	0.0755 (12)	0.0581 (11)	−0.0078 (10)	0.0206 (9)	0.0066 (9)
C17	0.0658 (13)	0.0817 (14)	0.0754 (15)	−0.0125 (11)	0.0249 (12)	0.0156 (11)
C18	0.0703 (13)	0.0621 (11)	0.0868 (16)	−0.0120 (10)	0.0200 (12)	0.0099 (11)
C19	0.0633 (12)	0.0571 (10)	0.0694 (13)	−0.0012 (9)	0.0148 (11)	−0.0001 (9)
C31	0.0562 (10)	0.0485 (9)	0.0479 (10)	−0.0066 (8)	0.0208 (9)	−0.0032 (7)
C32	0.0949 (15)	0.0591 (10)	0.0577 (12)	0.0070 (10)	0.0393 (12)	0.0031 (9)
C33	0.0916 (16)	0.0868 (14)	0.0614 (13)	0.0064 (12)	0.0424 (12)	−0.0036 (10)
C34	0.0772 (14)	0.0866 (14)	0.0653 (14)	0.0195 (12)	0.0261 (12)	−0.0074 (11)
C35	0.154 (3)	0.0787 (15)	0.117 (2)	0.0533 (16)	0.080 (2)	0.0320 (14)
C36	0.131 (2)	0.0701 (12)	0.0980 (18)	0.0313 (13)	0.0746 (17)	0.0290 (11)
C37	0.132 (2)	0.145 (2)	0.102 (2)	0.062 (2)	0.0533 (19)	−0.0064 (17)
C41	0.0504 (10)	0.0623 (10)	0.0505 (10)	0.0030 (8)	0.0175 (9)	−0.0039 (8)
C42	0.0582 (13)	0.1049 (17)	0.1071 (19)	−0.0202 (12)	0.0213 (13)	−0.0308 (14)
C43	0.0697 (14)	0.1080 (17)	0.0532 (13)	0.0155 (13)	0.0028 (11)	−0.0076 (11)
C44	0.0850 (15)	0.1026 (15)	0.0442 (12)	0.0253 (13)	0.0184 (11)	0.0128 (10)
C45	0.0728 (13)	0.0675 (11)	0.0522 (11)	0.0126 (10)	0.0302 (10)	0.0098 (9)
N1	0.0486 (8)	0.0615 (8)	0.0444 (8)	0.0015 (7)	0.0145 (7)	−0.0019 (6)
N2	0.0520 (9)	0.0787 (10)	0.0581 (10)	−0.0012 (8)	0.0116 (8)	−0.0140 (8)
N3	0.0557 (9)	0.0589 (8)	0.0449 (8)	−0.0018 (7)	0.0205 (7)	0.0019 (6)
N4	0.0559 (9)	0.0584 (8)	0.0507 (9)	0.0016 (7)	0.0154 (7)	−0.0038 (7)
O1	0.1314 (14)	0.0729 (9)	0.0757 (10)	−0.0015 (9)	0.0515 (10)	0.0191 (7)

### Geometric parameters (Å, º)

**Table:**

C1—N1	1.452 (2)	C16—C17	1.359 (3)
C1—H1A	0.9600	C16—H16	0.9300
C1—H1B	0.9600	C17—C18	1.387 (3)
C1—H1C	0.9600	C17—H17	0.9300
C2—N1	1.443 (2)	C18—C19	1.356 (3)
C2—C3	1.511 (2)	C18—H18	0.9300
C2—H2A	0.9700	C19—H19	0.9300
C2—H2B	0.9700	C31—C36	1.366 (2)
C3—C31	1.509 (2)	C31—C32	1.371 (2)
C3—C4	1.557 (2)	C32—C33	1.382 (3)
C3—H3	0.9800	C32—H32	0.9300
C4—C41	1.528 (2)	C33—C34	1.357 (3)
C4—C45	1.532 (2)	C33—H33	0.9300
C4—C5	1.607 (2)	C34—C35	1.360 (3)
C5—N1	1.462 (2)	C34—C37	1.515 (3)
C5—C12	1.521 (2)	C35—C36	1.372 (3)
C5—C6	1.528 (2)	C35—H35	0.9300
C6—C11	1.378 (2)	C36—H36	0.9300
C6—C7	1.391 (2)	C37—H37A	0.9600
C7—C8	1.382 (2)	C37—H37B	0.9600
C7—C13	1.456 (2)	C37—H37C	0.9600
C8—C9	1.371 (3)	C41—N2	1.456 (2)
C8—H8	0.9300	C41—H41A	0.9700
C9—C10	1.369 (3)	C41—H41B	0.9700
C9—H9	0.9300	C42—N2	1.459 (2)
C10—C11	1.384 (2)	C42—H42A	0.9600
C10—H10	0.9300	C42—H42B	0.9600
C11—H11	0.9300	C42—H42C	0.9600
C12—N3	1.3041 (19)	C43—N2	1.453 (3)
C12—C13	1.428 (2)	C43—C44	1.513 (3)
C13—N4	1.305 (2)	C43—H43A	0.9700
C14—N4	1.377 (2)	C43—H43B	0.9700
C14—C19	1.403 (2)	C44—C45	1.504 (3)
C14—C15	1.408 (2)	C44—H44A	0.9700
C15—N3	1.379 (2)	C44—H44B	0.9700
C15—C16	1.401 (2)	C45—O1	1.202 (2)
			
N1—C1—H1A	109.5	C19—C18—C17	120.68 (18)
N1—C1—H1B	109.5	C19—C18—H18	119.7
H1A—C1—H1B	109.5	C17—C18—H18	119.7
N1—C1—H1C	109.5	C18—C19—C14	120.28 (19)
H1A—C1—H1C	109.5	C18—C19—H19	119.9
H1B—C1—H1C	109.5	C14—C19—H19	119.9
N1—C2—C3	101.52 (13)	C36—C31—C32	115.86 (16)
N1—C2—H2A	111.5	C36—C31—C3	120.51 (15)
C3—C2—H2A	111.5	C32—C31—C3	123.56 (15)
N1—C2—H2B	111.5	C31—C32—C33	121.72 (17)
C3—C2—H2B	111.5	C31—C32—H32	119.1
H2A—C2—H2B	109.3	C33—C32—H32	119.1
C31—C3—C2	116.05 (13)	C34—C33—C32	122.13 (18)
C31—C3—C4	117.60 (14)	C34—C33—H33	118.9
C2—C3—C4	103.36 (12)	C32—C33—H33	118.9
C31—C3—H3	106.3	C33—C34—C35	115.91 (18)
C2—C3—H3	106.3	C33—C34—C37	121.7 (2)
C4—C3—H3	106.3	C35—C34—C37	122.4 (2)
C41—C4—C45	106.37 (14)	C34—C35—C36	122.7 (2)
C41—C4—C3	111.96 (12)	C34—C35—H35	118.7
C45—C4—C3	111.94 (13)	C36—C35—H35	118.7
C41—C4—C5	112.52 (12)	C31—C36—C35	121.73 (19)
C45—C4—C5	111.13 (12)	C31—C36—H36	119.1
C3—C4—C5	103.06 (12)	C35—C36—H36	119.1
N1—C5—C12	114.56 (13)	C34—C37—H37A	109.5
N1—C5—C6	109.26 (13)	C34—C37—H37B	109.5
C12—C5—C6	100.27 (12)	H37A—C37—H37B	109.5
N1—C5—C4	102.87 (12)	C34—C37—H37C	109.5
C12—C5—C4	113.56 (12)	H37A—C37—H37C	109.5
C6—C5—C4	116.79 (12)	H37B—C37—H37C	109.5
C11—C6—C7	120.22 (15)	N2—C41—C4	111.52 (13)
C11—C6—C5	127.58 (15)	N2—C41—H41A	109.3
C7—C6—C5	112.10 (14)	C4—C41—H41A	109.3
C8—C7—C6	120.69 (16)	N2—C41—H41B	109.3
C8—C7—C13	130.67 (16)	C4—C41—H41B	109.3
C6—C7—C13	108.49 (13)	H41A—C41—H41B	108.0
C9—C8—C7	118.62 (18)	N2—C42—H42A	109.5
C9—C8—H8	120.7	N2—C42—H42B	109.5
C7—C8—H8	120.7	H42A—C42—H42B	109.5
C10—C9—C8	120.89 (17)	N2—C42—H42C	109.5
C10—C9—H9	119.6	H42A—C42—H42C	109.5
C8—C9—H9	119.6	H42B—C42—H42C	109.5
C9—C10—C11	121.17 (18)	N2—C43—C44	110.57 (17)
C9—C10—H10	119.4	N2—C43—H43A	109.5
C11—C10—H10	119.4	C44—C43—H43A	109.5
C6—C11—C10	118.38 (17)	N2—C43—H43B	109.5
C6—C11—H11	120.8	C44—C43—H43B	109.5
C10—C11—H11	120.8	H43A—C43—H43B	108.1
N3—C12—C13	123.00 (14)	C45—C44—C43	113.41 (16)
N3—C12—C5	125.97 (14)	C45—C44—H44A	108.9
C13—C12—C5	110.83 (13)	C43—C44—H44A	108.9
N4—C13—C12	124.14 (15)	C45—C44—H44B	108.9
N4—C13—C7	127.68 (15)	C43—C44—H44B	108.9
C12—C13—C7	108.10 (14)	H44A—C44—H44B	107.7
N4—C14—C19	118.75 (16)	O1—C45—C44	120.93 (18)
N4—C14—C15	122.30 (15)	O1—C45—C4	122.54 (18)
C19—C14—C15	118.95 (16)	C44—C45—C4	116.52 (17)
N3—C15—C16	118.83 (16)	C2—N1—C1	116.41 (13)
N3—C15—C14	121.84 (15)	C2—N1—C5	107.90 (12)
C16—C15—C14	119.32 (16)	C1—N1—C5	115.85 (13)
C17—C16—C15	120.02 (19)	C43—N2—C41	109.14 (15)
C17—C16—H16	120.0	C43—N2—C42	111.38 (17)
C15—C16—H16	120.0	C41—N2—C42	110.91 (15)
C16—C17—C18	120.73 (19)	C12—N3—C15	114.63 (14)
C16—C17—H17	119.6	C13—N4—C14	113.89 (14)
C18—C17—H17	119.6		
			
N1—C2—C3—C31	−172.10 (13)	C14—C15—C16—C17	0.6 (3)
N1—C2—C3—C4	−41.85 (15)	C15—C16—C17—C18	0.6 (3)
C31—C3—C4—C41	30.84 (19)	C16—C17—C18—C19	−0.9 (3)
C2—C3—C4—C41	−98.47 (15)	C17—C18—C19—C14	0.0 (3)
C31—C3—C4—C45	−88.50 (17)	N4—C14—C19—C18	−177.93 (16)
C2—C3—C4—C45	142.19 (14)	C15—C14—C19—C18	1.1 (3)
C31—C3—C4—C5	152.00 (13)	C2—C3—C31—C36	−156.72 (18)
C2—C3—C4—C5	22.69 (14)	C4—C3—C31—C36	80.2 (2)
C41—C4—C5—N1	124.83 (13)	C2—C3—C31—C32	20.2 (2)
C45—C4—C5—N1	−116.00 (15)	C4—C3—C31—C32	−102.9 (2)
C3—C4—C5—N1	4.05 (14)	C36—C31—C32—C33	0.3 (3)
C41—C4—C5—C12	0.45 (18)	C3—C31—C32—C33	−176.66 (18)
C45—C4—C5—C12	119.62 (15)	C31—C32—C33—C34	0.2 (3)
C3—C4—C5—C12	−120.33 (13)	C32—C33—C34—C35	−0.3 (3)
C41—C4—C5—C6	−115.54 (15)	C32—C33—C34—C37	179.3 (2)
C45—C4—C5—C6	3.63 (19)	C33—C34—C35—C36	0.0 (4)
C3—C4—C5—C6	123.68 (14)	C37—C34—C35—C36	−179.6 (3)
N1—C5—C6—C11	51.5 (2)	C32—C31—C36—C35	−0.7 (3)
C12—C5—C6—C11	172.17 (17)	C3—C31—C36—C35	176.4 (2)
C4—C5—C6—C11	−64.7 (2)	C34—C35—C36—C31	0.6 (4)
N1—C5—C6—C7	−125.00 (15)	C45—C4—C41—N2	−58.10 (18)
C12—C5—C6—C7	−4.29 (17)	C3—C4—C41—N2	179.34 (13)
C4—C5—C6—C7	118.85 (16)	C5—C4—C41—N2	63.81 (17)
C11—C6—C7—C8	1.8 (3)	N2—C43—C44—C45	48.0 (2)
C5—C6—C7—C8	178.55 (15)	C43—C44—C45—O1	137.3 (2)
C11—C6—C7—C13	−174.11 (15)	C43—C44—C45—C4	−41.7 (2)
C5—C6—C7—C13	2.64 (19)	C41—C4—C45—O1	−134.21 (18)
C6—C7—C8—C9	−0.5 (3)	C3—C4—C45—O1	−11.6 (2)
C13—C7—C8—C9	174.35 (18)	C5—C4—C45—O1	103.01 (19)
C7—C8—C9—C10	−0.9 (3)	C41—C4—C45—C44	44.83 (19)
C8—C9—C10—C11	1.0 (3)	C3—C4—C45—C44	167.41 (15)
C7—C6—C11—C10	−1.6 (3)	C5—C4—C45—C44	−77.95 (19)
C5—C6—C11—C10	−177.83 (17)	C3—C2—N1—C1	179.32 (13)
C9—C10—C11—C6	0.2 (3)	C3—C2—N1—C5	47.10 (16)
N1—C5—C12—N3	−53.7 (2)	C12—C5—N1—C2	92.15 (15)
C6—C5—C12—N3	−170.56 (15)	C6—C5—N1—C2	−156.30 (13)
C4—C5—C12—N3	64.1 (2)	C4—C5—N1—C2	−31.57 (15)
N1—C5—C12—C13	121.29 (15)	C12—C5—N1—C1	−40.37 (18)
C6—C5—C12—C13	4.46 (17)	C6—C5—N1—C1	71.18 (16)
C4—C5—C12—C13	−120.91 (14)	C4—C5—N1—C1	−164.10 (13)
N3—C12—C13—N4	−5.0 (3)	C44—C43—N2—C41	−61.5 (2)
C5—C12—C13—N4	179.81 (15)	C44—C43—N2—C42	175.73 (17)
N3—C12—C13—C7	171.94 (15)	C4—C41—N2—C43	69.31 (18)
C5—C12—C13—C7	−3.26 (18)	C4—C41—N2—C42	−167.62 (16)
C8—C7—C13—N4	1.8 (3)	C13—C12—N3—C15	3.6 (2)
C6—C7—C13—N4	177.18 (16)	C5—C12—N3—C15	178.01 (14)
C8—C7—C13—C12	−174.98 (18)	C16—C15—N3—C12	179.20 (15)
C6—C7—C13—C12	0.39 (19)	C14—C15—N3—C12	0.5 (2)
N4—C14—C15—N3	−3.7 (3)	C12—C13—N4—C14	1.6 (2)
C19—C14—C15—N3	177.29 (15)	C7—C13—N4—C14	−174.70 (15)
N4—C14—C15—C16	177.59 (16)	C19—C14—N4—C13	−178.54 (15)
C19—C14—C15—C16	−1.4 (2)	C15—C14—N4—C13	2.4 (2)
N3—C15—C16—C17	−178.18 (16)		

### Hydrogen-bond geometry (Å, º)

**Table:**

*D*—H···*A*	*D*—H	H···*A*	*D*···*A*	*D*—H···*A*
C3—H3···O1	0.98	2.36	2.804 (2)	107
C41—H41*B*···N3	0.97	2.39	2.980 (2)	119
C11—H11···*Cg*1^i^	0.93	2.94	3.692 (2)	139

Symmetry code: (i) *x*, −*y*−1, *z*−1/2.
